# Fracture Resistance of Endodontically Treated Teeth with Different Perforation Diameters: An In Vitro Analysis

**DOI:** 10.3390/dj14010012

**Published:** 2026-01-01

**Authors:** Flora Kakoura, Kleoniki Lyroudia, Nikolaos Economides, Dimitrios Dimitriadis, Georgios Mikrogeorgis

**Affiliations:** 1 Department of Endodontology, School of Dentistry, Aristotle University of Thessaloniki, 54124 Thessaloniki, Greece; fkakour@dent.auth.gr (F.K.); econom@dent.auth.gr (N.E.); 2 Department of Economics, School of Economic and Political Sciences, Aristotle University of Thessaloniki, 54124 Thessaloniki, Greece

**Keywords:** fracture resistance, fracture strength, root perforation, perforation diameter, root fracture

## Abstract

**Objectives**: The aim of this study was to investigate the relationship between the diameter of iatrogenic root perforations and the fracture resistance (FR) of endodontically treated teeth. **Methods**: Sixty-five single-rooted teeth were sectioned at 13 mm from the anatomic apex. Their weight and the mesiodistal (MD) and buccolingual (BL) dimensions were recorded in order to ensure their allocation into five homogeneous groups (N = 13); Group 1 (control group): teeth remained intact, Group 2: teeth were instrumented but not perforated, Group 3: teeth were instrumented and perforated with a 2.1 mm bur, Group 4: teeth were instrumented and perforated with a 1 mm bur, and Group 5: teeth were instrumented and perforated with a 0.5 mm bur. All perforations were created at the same spot in the buccal surface of the roots. To further standardize the defects, an apparatus ensured that the cutting bur was positioned with a steady angle of 105° to the external root surface. A universal testing machine was used for fracture testing by applying a compressive vertical load at a speed of 1 mm/min until root fracture. The recorded forces were statistically analyzed with one-way analysis of variance (ANOVA) and post hoc Tukey test. **Results**: The mean fracture load was 342.68 ± 146.45 N for Group 1, 322.96 ± 98.62 N for Group 2, 214.65 ± 71.32 N for Group 3, 212.66 ± 77.89 N for Group 4, and 307.14 ± 109.16 N for Group 5. However, only the differences between groups 1–3 and 1–4 were statistically significant (*p* < 0.05). **Conclusions**: The teeth with 2.1 mm and 1 mm perforations were found to have significantly decreased FR.

## 1. Introduction

Root canal therapy is the treatment of choice in cases of irreversible pulp inflammation or apical periodontitis [1]. Due to the increased prevalence of endodontic disease [2], the global percentage of people with at least one endodontic therapy is estimated to be up to 55.7% [3]. The diversity in the anatomy of root canals poses a challenge for the clinician during access cavity and chemomechanical preparation of the root canal space. The occurrence of procedural accidents, such as ledge formation, canal transportation and root perforation, is a common mishap [4].

Intraoperative perforation of the root refers to an iatrogenic defect created during endodontic procedures, resulting in an unintended communication between the root canal system and the surrounding periodontal tissues. This complication may arise from improper use of dental burs and drills, misdirected hand or rotary endodontic files, and predisposing factors such as atypical tooth positioning or root canal calcification [5]. Retrospective studies estimate the prevalence of mechanical root perforations from 2.3% [6] to 2.7% [7].

According to Fuss and Trope [8], the prognosis of a perforation is highly dependent on the bacterial contamination of the perforation site. Thus, the time interval between the occurrence of the perforation and its management is of paramount importance. Likewise, the size and site of the perforation significantly influence the likelihood of favorable healing. Small communications are associated with less periodontal damage and easier sealing. On the contrary, contamination is more probable when the perforation is proximal to the level of the crestal bone, as oral bacteria can migrate easily through the sulcus to the defect [8].

The loss of dental structure is linked to higher susceptibility to root fracture [9], affecting the survival outcome of the weakened tooth [10]. The fracture resistance (FR) of teeth with severe non-carious cervical defects or simulated external and internal resorption cavities has been previously assessed [11,12,13,14,15,16,17]. Like in pathologic processes, dentin loss in cases of iatrogenic perforations can be extensive. To date, there is no study investigating the impact of the size of root perforation on fracture strength, and subsequently on the prognosis, of endodontically treated teeth. Therefore, the objective of this in vitro study was to assess the influence of the diameter of standardized perforations in the FR of endodontically treated roots. The null hypothesis was that, regardless of the diameter, all perforations affect the FR.

## 2. Materials and Methods

### 2.1. Sample Selection and Preparation

This study was conducted in accordance with the Declaration of Helsinki and received ethical approval from the Ethics Committee of the Aristotle University of Thessaloniki Dental School (protocol no. 126/14-07-2021). All experimental procedures were carried out at the School of Dentistry, Faculty of Health Sciences, Aristotle University of Thessaloniki, Greece.

The sample size for the present study was determined based on a pilot study, which yielded an effect size of 0.572. A power level of 0.95 (1 − β) and a significance level of α = 0.05 were used. The data were analyzed using a one-way analysis of variance (ANOVA) conducted with G*Power 3.1 software for Windows. The optimal sample size was calculated to be 65 statistical units.

A total of 65 single-rooted teeth extracted for periodontal reasons were selected for the study. Only intact teeth free from caries, cracks, and any signs of internal or external resorption were included. All samples were cleaned and stored in 0.1% thymol solution until use and for no longer than two months [18]. Teeth were inspected for cracks with a stereomicroscope (Stemi 2000C, Carl Zeiss, Oberkochen, Germany) under ×10 magnification and radiographed buccolingually and mesiodistally to ensure the presence of a single root canal free from calcification or resorption. All specimens were precisely sectioned at 13 mm from the anatomical apex with the aid of a diamond wafering blade (Isomet Blade, Buehler, Waukegan, IL, USA) under water cooling.

Thereafter, the teeth were randomly allocated equally into five groups. The buccolingual (BL) and mesiodistal (MD) dimensions, as well as the weight, of each root were recorded with the aid of a digital caliper and a precision scale, respectively. The dimensional homogeneity of the specimens within and between groups was statistically evaluated, as described in Section 2.3.

**Figure 1:**
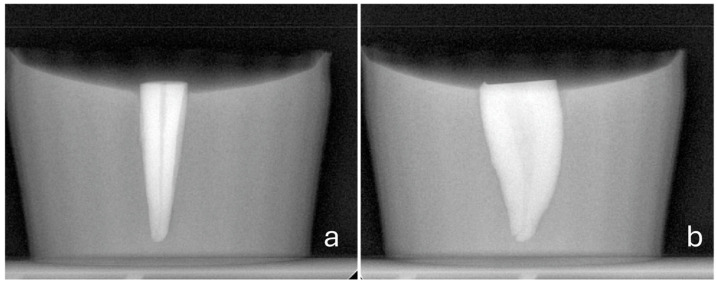
(**a**) Buccolingual (BL) and (**b**) mesiodistal (MD) radiographs confirming the vertical positioning of the roots in the acrylic blocks.

All roots were covered with a one-thickness layer lead foil and placed vertically into silicon molds filled with auto-polymerizing acrylic resin (BMS 015 powder/liquid; BMS Dental), exposing 1 mm of the coronal part. Then, the surrounding foil was removed, and the acrylic blocks were radiographed using the paralleling technique at BL and MD angulations to ensure the vertical placement of the teeth (Figure 1) [19].

Thirteen teeth served as the negative control group without any intervention (Group 1). The working length of the remaining 52 root canals was negotiated with a #10-K hand file (Dentsply, Maillefer, Ballaigues, Switzerland) and determined 1 mm short of the apex. Using rotary files (ESX^®^ Instrumentation System; Brasseler USA, Savannah, GA, USA), all roots were instrumented up to a master apical size of #35/.04. Mechanical shaping was combined with meticulous intermittent irrigation with 3 mL 2.5% sodium hypochlorite. After instrumentation, all specimens were re-examined under the stereomicroscope for the detection of newly formed cracks or craze lines [20]. Apart from chemomechanical preparation, the teeth included in Group 2 did not receive any further processing.

**Figure 2:**
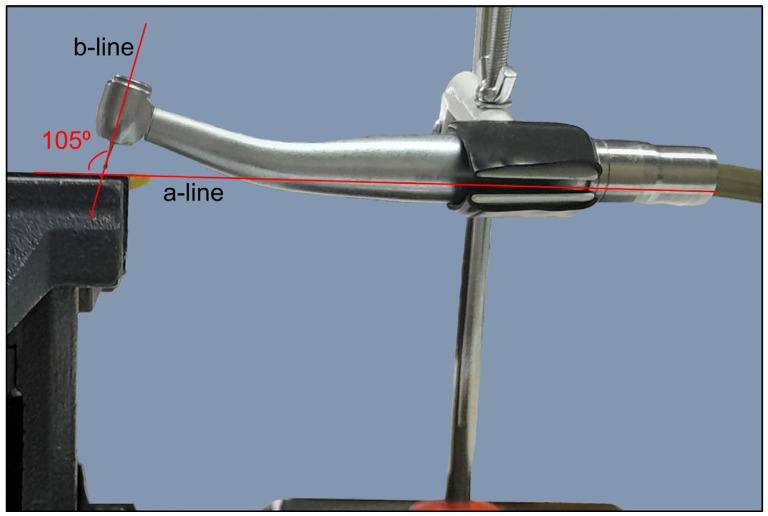
Part of the experimental setup depicting the steady relationship between the buccal surface of the roots (a-line) and the dental drilling device (b-line). For clarity, the background of the image was digitally removed using Adobe Photoshop CC (Adobe Systems Inc., San Jose, CA, USA).

**Figure 3:**
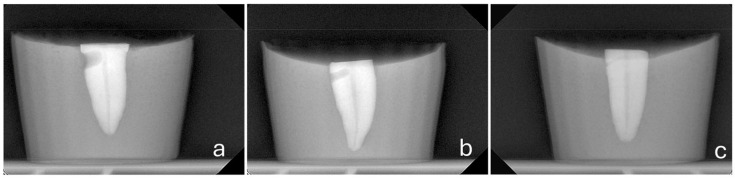
MD radiographs of representative samples of Group 3 (**a**), Group 4 (**b**) and Group 5 (**c**), showing simulated perforations of 2.1 mm, 1.0 mm, and 0.5 mm in diameter, respectively.

To ensure consistent perforations, the experiment incorporated the following parameters:

- A consistent perforation diameter. High-speed round burs with diameters of 2.1 mm, 1 mm and 0.5 mm (SS White, Lakewood, NJ, USA) were used for the Groups 3, 4 and 5, respectively. Each bur was used once and then discarded.
- A steady direction of perforation. Teeth were secured in a vise with their buccal surfaces parallel to the horizontal plane. The high-speed handpiece was also fixed in a horizontal position using a custom-made apparatus that allowed only downward vertical movement. Buccal diagonal perforations, starting 3 mm apical to the coronal surface of the teeth, were created under water cooling with a steady angle of 105° between the external surface of the root and the shank of the bur (Figure 2 and Figure 3). A #35-K stainless steel hand file (Dentsply, Maillefer, Ballaigues, Switzerland) inside the root canal prevented the overextension of the perforation to the lingual surface of the root.

All perforations were performed by the same operator in order to minimize variability and ensure consistency across groups.

### 2.2. Fracture Resistance Testing

**Figure 4:**
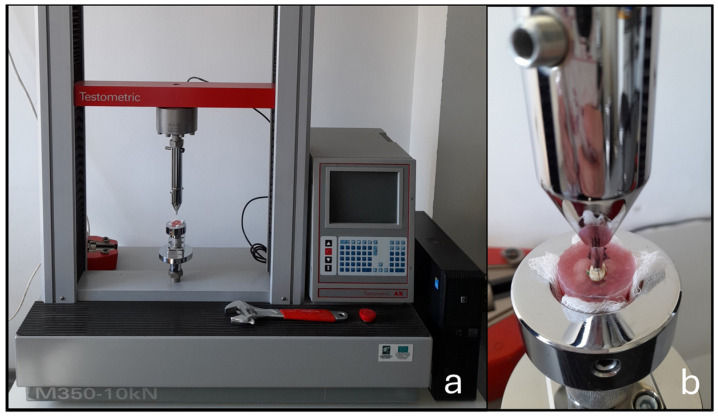
Part of the experimental setup is shown, illustrating a sample mounted on the universal testing machine (**a**). A compressive vertical load was applied until root fracture occurred (**b**). The force at the moment of fracture was recorded in newtons (N).

The roots were covered with a thin layer of vinyl polysiloxane impression material (3M Express, 3M Espe, 3M, Saint Paul, MN, USA) and placed directly into their acrylic blocks to simulate the periodontal ligament [21]. After setting, the excess impression material was removed from the perforations with the help of a surgical blade. The teeth, embedded in their artificial sockets, were then mounted in a universal testing machine (Testometric M350-10 KN; Linkon Close, Rochdale, UK). A stainless steel probe, ending in a 0.5 mm diameter 60° conical tip, was aligned above the canal orifice and a vertical compressive load with a speed of 1 mm/min was applied until fracture (Figure 4). The force at which the roots fractured was recorded in newtons (N).

### 2.3. Statistical Analysis

The homogeneity of the specimen dimensions within each group was assessed. Specifically, a box plot analysis was conducted to identify potential outliers; none were detected. The Shapiro–Wilk test supported the assumption of normality for all variables involved (*p* > 0.05). Given that the normality assumption was met, a one-way ANOVA was performed to examine statistically significant differences among the groups.

Regarding the force to fracture (N), box plot analysis was used to investigate the existence of outliers within groups. No outliers were found. The assumption of normal distribution for the force variable was also supported through the Shapiro–Wilk test (*p* > 0.05). The one-way ANOVA procedure was used in order to examine statistically significant differences among groups. Finally, pairwise multiple comparison procedures were performed using Tukey’s HSD approach. All analyses were conducted using IBM SPSS version 25 software, with all tests performed at a 5% significance level.

## 3. Results

The mean and standard deviation values of the dimensions for all groups, along with the results of the ANOVA, are presented in Table 1. No statistically significant differences were observed among the groups (*p* > 0.05), thereby supporting their dimensional homogeneity.

The mean and standard deviation values of the fracture load were 342.68 ± 146.45 N for Group 1, 322.96 ± 98.62 N for Group 2, 214.65 ± 71.32 N for Group 3, 212.66 ± 77.89 N for Group 4, and 307.14 ± 109.16 N for Group 5. The one-way ANOVA revealed a statistically significant difference among the groups with respect to the load-to-fracture values (*p* < 0.05) (Table 1). To further investigate the group differences identified by the one-way ANOVA, Tukey’s HSD post hoc test was applied for all pairwise comparisons. The results demonstrated that the control group (Group 1) exhibited significantly higher FR compared to Group 3 (*p* = 0.022) and Group 4 (*p* = 0.019). No other statistically significant differences were observed among the remaining groups. These findings indicate that only medium and large perforation diameters produced a measurable reduction in FR, whereas the smallest perforation size (0.5 mm) did not significantly alter the mechanical behavior of the roots.

## 4. Discussion

The amount of root tissue that remains after root canal treatment is one of the most influential factors affecting the strength of teeth [22]. To date, no studies have specifically investigated the impact of perforation size on the FR of endodontically treated teeth. Existing research on load-to-fracture in teeth with radicular defects has been limited to cases involving pathological destruction of radicular dentin—such as external and internal root resorption—typically repaired with various restorative materials [11,12,13,14,15,16,17]. Nevertheless, certain studies offer comparable data that may serve as references for our findings [11,13,14].

The standardization of the study and test parameters is of paramount importance in FR testing using natural teeth [23]. In previous studies, information regarding the method of radicular defect formation is limited [11,13,14], and it is presumed that the defects were manually prepared using a high-speed handpiece in the absence of standardized guidance. However, the creation of defects with controlled dimensions across all study groups enhances the reliability of comparisons. In this in vitro experiment, the simulated endodontic perforations were standardized in two key aspects. A consistent defect diameter was achieved using ISO-standard dental burs with verified dimensions. Each perforation was created through a single, controlled downward movement of a securely mounted high-speed handpiece, minimizing unintended bur drift and ensuring cavities with smooth, well-defined outlines. Additionally, the orientation of the handpiece facilitated the formation of a diagonal perforation with a consistent angle relative to the external root surface across all specimens. This drilling approach simulates clinical conditions, as most perforations occurring during access cavity preparation or mechanical instrumentation tend to follow a diagonal apical trajectory.

Another important parameter to consider is the natural variability in tooth anatomy among individuals. Significant differences in root dimensions may influence the results and should be acknowledged as a potential source of variability in the study outcomes [23]. In most FR experiments, information regarding the dimensions of the specimens is inadequately described, potentially limiting the reproducibility of the findings [11,13,14,17]. To minimize dimensional bias, the coronal BL and MD dimensions, along with the weight of each specimen, were recorded and subjected to statistical analysis. This allowed for the formation of homogeneous groups, thereby enhancing the reliability and comparability of the results across experimental conditions. Moreover, storage conditions, including both the medium and duration, were carefully considered. The teeth were stored in 0.1% thymol solution for no longer than two months, a protocol shown not to significantly affect the mechanical properties of dentin, making it suitable for in vitro mechanical testing [18].

Currently, the influence of canal taper and apical preparation size on the reduction in FR remains a topic of ongoing debate in the literature [24]. In light of the contrasting findings, an additional group of teeth that were solely instrumented (Group 2) was included in the study. Since the FR observed between the intact and instrumented teeth was similar, it was inferred that this test parameter did not substantially increase the risk of fracture. The apical size of #35/0.04 was chosen to balance adequate cleaning with preservation of radicular dentin. It is acknowledged that results may differ with other preparation sizes, and conclusions are therefore limited to this preparation protocol.

The simulation of the periodontal ligament and bone support is recommended during monotonic tests [21,25]. In this study, a polymethylmethacrylate acrylic resin resembled the alveolar process, while a vinyl polysiloxane impression material was employed to replicate the periodontal ligament and mimic the natural mobility of the tooth within the alveolar socket [21]. Furthermore, the design of the fracture test probe allowed for precise placement at the entrance of the root canal, ensuring the application of a controlled vertical force. While vertical loading does not fully replicate the complex forces encountered in clinical conditions, the standardized loading angle facilitated uniform testing conditions and enabled a reliable comparison of FR among the experimental groups. Forces applied at varying orientations and with different types of probes—such as spherical [11,12,14,15,16,17] and wedge-shaped instruments [13]—have been examined in previous studies. According to one study, the type of probe used to apply compressive loads during FR testing may significantly influence the measured force values [26].

To the best of our knowledge, no other studies currently investigate the FR of teeth with small (0.5 mm) and medium (1 mm) perforations, or directly compare them to groups with either no defects or large (≥2 mm) perforations. Our results indicate a difference in the FR between intact teeth (Group 1) and teeth with large or medium perforations (Groups 3 & 4, respectively). Consistent with our findings, premolar roots with simulated internal resorption cavities measuring 1.4 mm [11] and 2.3 mm [14] were found to be more susceptible to fracture compared to intact teeth. In the study by Romeo, mandibular anterior teeth were used to create external resorption cavities measuring 2.9 mm in diameter at the cementoenamel junction. Similarly, the maximum load capacity of the intact teeth was significantly higher than that of teeth with large defects [13]. The substantial methodological differences in other studies investigating the fracture strength of teeth with root defects limit the ability to directly compare their results with ours. Specifically, in one study that measured the load-to-fracture of roots with simulated resorptive cavities measuring 1 mm in diameter and 0.5 mm in depth, no control group of intact teeth was included for comparison, limiting the interpretability of the results [17]. Moreover, other studies simulated the internal resorption cavities by enlarging the coronal interior of the root canal without creating tunnel-like defects that extend to the external root surface [12,16]. Regarding the small perforation group (Group 5), there is a lack of comparable data in the existing literature. In the present study, the presence of small perforation defects did not significantly affect the FR of the teeth. The fact that 0.5 mm perforations did not significantly affect fracture resistance may be explained by the limited structural loss they induce. It should be noted that the effect of perforation location (apical, middle, or cervical third) was not examined here but could meaningfully alter outcomes.

This investigation was conducted under strictly controlled in vitro conditions, which inevitably differ from the complex biological and mechanical environment of the oral cavity. Consequently, the results of the present study cannot be directly extrapolated to clinical scenarios, as they do not account for the influence of functional loading, temperature variations, saliva, and the dynamic behavior of the periodontal ligament. Nevertheless, in vitro experiments such as the present study provide essential baseline data for understanding how structural variables—such as perforation diameter—affect the mechanical integrity of endodontically treated teeth. These findings can therefore serve as a foundation for future in vivo and clinical research aimed at validating and expanding the present observations.

## 5. Conclusions

Within the limitations of this in vitro study, it can be concluded that small perforations created during root canal treatment did not significantly affect the fracture strength of the roots. In contrast, perforations ≥1 mm in size resulted in a notable weakening of the tooth structure. This finding should be taken into consideration after the completion of endodontic therapy, particularly during the final restoration of the tooth. Careful planning of the restorative procedure is essential, as teeth with larger perforations may require additional reinforcement to prevent fracture under functional loads. Further research is necessary to assess the applicability of these findings to clinical scenarios, particularly through the investigation of additional parameters such as oblique loading angles and perforations located in different root thirds.

## Figures and Tables

**Table 1:** Mean and standard deviation values of the BL & MD dimensions, the weight and the fracture resistance (FR) force of the experimental groups.

	Group 1	Group 2	Group 3	Group 4	Group 5	ANOVA (*p*-Values)
**BL (mm)**	7.82 ± 0.55	7.86 ± 0.60	7.82 ± 0.59	7.70 ± 0.56	7.71 ± 0.59	0.933
**MD (mm)**	4.62 ±0.15	4.63 ± 0.24	4.68 ± 0.15	4.72 ± 0.29	4.51 ± 0.18	0.136
**Weight (g)**	0.45 ± 0.02	0.47 ± 0.10	0.44 ± 0.04	0.44 ± 0.05	0.46 ± 0.04	0.550
**Force (N)**	342.68 ± 146.45	322.96 ± 98.62	214.65 ± 71.32	212.66 ± 77.89	307.14 ± 109.16	0.003 **

mm = millimeter, g = gram, N = newtons. ** Significant at 0.05 level.

## Data Availability

The datasets generated and analyzed during the current study are not publicly available due to their inclusion in a PhD project in progress, but they are available from the corresponding author upon reasonable request.

## Abbreviations

The following abbreviations are used in this manuscript:

FR	fracture resistance
ANOVA	analysis of variance
BL	buccolingual
MD	mesiodistal
N	newtons
mm	millimeter
g	gram
